# Molecular Characterization of Rotavirus Gastroenteritis Strains, Iraqi Kurdistan

**DOI:** 10.3201/eid1205.051422

**Published:** 2006-05

**Authors:** Herish M. Ahmed, J. Brian S. Coulter, Osamu Nakagomi, C.A. Hart, Jamal M. Zaki, Abas A. Al-Rabaty, Winifred Dove, Nigel A. Cunliffe

**Affiliations:** * Liverpool School of Tropical Medicine, Liverpool, UK;; † University of Liverpool, Liverpool, UK;; ‡ Erbil Paediatric Hospital, Erbil, Iraqi Kurdistan;; § Nagasaki University, Nagasaki, Japan

**Keywords:** Rotavirus, genotypes, diarrhea, children, Iraqi Kurdistan

## Abstract

Of 260 children with acute diarrhea in Erbil, Iraqi Kurdistan, 96 (37%) were infected with rotavirus. Reverse transcription–polymerase chain reaction identified G1, G4, G2, G9, P[8], P[6], and P[4] as the most common genotypes. Eight G/P combinations were found, but P[8]G1 and P[4]G2 accounted for >50% of the strains.

Rotavirus is the single most important cause of severe gastroenteritis in young children throughout the world. Globally, an estimated 702,000 children die each year due to rotavirus diarrhea (*1*). This large impact of rotavirus disease has speeded the development of rotavirus vaccines, and 2 live, attenuated rotavirus vaccines are expected to be available for global use within the next few years (*1*). Therefore, determining the prevalence and types of rotaviruses within regions is essential to prepare for introducing a vaccine.

Rotavirus, a member of the family *Reoviridae*, has a triple-layered capsid that contains 11 segments of double-stranded genomic RNA. While protective immunity against rotavirus infection is not completely understood, serotype-specific immunity is believed to play a major role (*1*). Rotavirus serotypes are defined by genome segment 4 for the P (protease-sensitive protein) type and by genome segment 9 (or 7 or 8, depending on the strain) for the G (glycoprotein) type. Fourteen G types exist, of which G1–G4 are commonly found in children with diarrhea, but a recent increase in the detection of serotype G8 and G9 strains has captured considerable attention (*2**–**4*). While >24 P types have been reported in the literature, only P[4], P[6], and P[8] are commonly found among human rotaviruses (*1**–**3*).

In Iraq, the death rate in children <5 years of age was reported to be 130/1,000 for boys and 120/1,000 for girls in 2003 (*5*). Diarrhea is a major cause of illness and death in Iraqi children; however, little information exists about the origin of childhood diarrhea. Only a single study showed that rotavirus accounted for 24% of acute diarrhea in hospitalized children in Basrah (*6*).

## The Study

This descriptive, cross-sectional study of 6 weeks' duration was undertaken at Erbil Paediatric Hospital in Iraqi Kurdistan between March and May 2005. The study recruited 260 children from 1 month to 5 years of age who were admitted with acute diarrhea (defined as the passage of watery or loose stools >3 times per day for <2 weeks' duration). Basic demographic, epidemiologic, and clinical information were collected prospectively, according to a pro forma. Ethical approval for the research was obtained from the review boards of the Liverpool School of Tropical Medicine and Erbil Paediatric Hospital. The hospital serves a population of ≈1.5 million, and ≈3,116 births per month occur in this population.

A commercial enzyme-linked immunosorbent assay (ELISA) was used to detect rotavirus antigen (Rotaclone, Meridian Diagnostics, Cincinnati, OH, USA). Stool samples were then stored frozen in the laboratory of the study hospital until they were transported to Liverpool for rotavirus genotyping and electropherotyping. All samples (66) with an absorbance equal to or greater than the positive control for the ELISA were subjected to genotyping. Rotavirus genomic RNA was extracted with guanidine isothiocyanate, followed by adsorption to and elution from silica particles according to the method described by Gentsch et al. (*7*). The purified RNA was then used to determine the P type and G type of rotavirus present in the stool specimens by reverse transcription-polymerase chain reaction as described by Gentsch et al. (*7*) and by Gouvea et al. (*8*). Rotavirus electropherotypes were determined by polyacrylamide gel electrophoresis according to the method described by Koshimura et al. (*9*), with some modifications.

Of 260 stool specimens tested by ELISA, 96 (37%) were positive for rotavirus. Rotavirus-positive patients had a mean age (SD) of 9.3 (8.5) months compared to 11.1 (10.1) months in the rotavirus-negative patients. These results suggest that rotavirus positive cases were slightly younger, although the difference was not statistically significant (p = 0.14). Rotavirus-positive patients were similar to rotavirus-negative patients in most of the epidemiologic and clinical characteristics (data not shown). However, rotavirus-positive patients were more likely to exhibit vomiting and have a shorter duration of diarrhea (p<0.01 for both analyses).

Of the 66 rotavirus strains that underwent molecular characterization, 25 (38%) were G1, 11 (17%) were G2, 13 (20%) were G4, and 7 (11%) were G9. Four (6%) were mixed infections (3 G1/G2, 1 G2/G4), and 6 (9%) were G nontypeable. A total of 7 (11%) were P[4], 10 (15%) were P[6], and 45 (68%) were P[8]. One showed mixed P[4] and P[8] genotypes (mixed with G1/G2), and 3 (5%) were P nontypeable. None of the rotaviruses was both G and P nontypeable.

A total of 8 different P and G genotype combinations were detected (Table). The most common combinations were P[8]G1 (19, 33%), P[8]G4 (12, 21%), P[4]G2 (6, 11%), P[6]G1 (6, 11%), and P[8]G9 (6, 11%). The unusual combination of P[6]G9 was detected in 1 of the patients.

**Table:** Rotavirus genotypes and electropherotypes*

Genotype	No.(%) fully typeable strains	Electropherotype†
P[4]G2	8 (15)	Short (7/8)
P[6]G1	6 (11)	Long (5/6)
P[6]G4	1 (2)	ND
P[6]G9	1 (2)	Long
P[8]G1	19 (33)	Long (13/19)
P[8]G4	12 (21)	Long (12/12)
P[8]G9	6 (11)	Long (4/6); short (1/6)
P[6]GNT	2	Long (2/2)
P[8]GNT	4	Long (2/4)
P[NT]G2	3	Short (3/3)

*Four rotavirus infections were mixed: P[8]G1/G2 (2), P[4]G2/G4 and P[4]/[8]G1/G2. †Indicates number of strains electropherotypeable in the genotype combination; ND, not determined.

An electropherotype was obtained for 50 of the 66 genotyped strains. Of these, 11 (22%) had a short electropherotype, and 39 (78%) had a long electropherotype (Table). Most of the short electropherotypes were the expected G2 strains; however, 1 strain (P[8]G9) also had a short electropherotype.

## Conclusions

The only other study of viral gastroenteritis from Iraq (Basrah in the south) demonstrated that 24% of children with acute gastroenteritis were infected with rotavirus (*6*). This figure is somewhat lower than the 37% detection rate in our study. Moreover, the prevalence we found is similar to those reported from neighboring countries such as Iran (35%) (*10*), Jordan (33%) (*11*), Kuwait (40%) (*12*), and Turkey (37%) (*13*). However, our study was undertaken over a 6-week period from the end of March to the beginning of May 2005. No information is available on the seasonal prevalence of rotavirus infection in Iraq, and a longer study is warranted to determine the true prevalence of rotavirus infection and its seasonality in northern Iraq. However, the peaks of rotavirus infection in Iran, Kuwait, and Turkey were February–March, March–May, and December, respectively (*10**,**12**,**13*). More than 75% of our cases of rotavirus diarrhea occurred in children <1 year of age, with an overall mean age of slightly more than 9 months. This pattern is similar to that in many developing countries. In Jordan the mean age of children with rotavirus diarrhea was 7.2 months (*10*). However, in other countries in the region the distribution was different; 30% of the infants with rotavirus in Iran were <1 year of age (*10**,**12*), 50% in Kuwait were <1 year of age, and 63% in Turkey were <2 years of age (*13*).

Although this study period was brief, we detected a variety of rotavirus strains. Four of the major global human rotavirus genotypes (G1, G2, G4, G9) were detected, as were each of the major P genotypes (P[4], P[6], P[8]). In Iran, in a study undertaken in 2001 and 2002, only G1 and G2 rotaviruses were detected, and the only P types were P[4] and P[8] (*10*), and in Turkey over a 2-year period (2000–2002), G types G1–G4 and G9, as well as each of the 3 major human P types were found (*14*). In Iraq, the combinations P[8]G1 and P[8]G4 accounted for >50% of the strains of rotavirus. In Iran, P[8]G1 accounted for 95% of the strains, but P[8]G4 was not detected (*10*). In Turkey, P[8]G4 (42%) and P[8]G1 (27%) accounted for more than two thirds of the strains (*14*). G3 rotaviruses were not detected in Iraq or Iran, and in Turkey only 1 of the 65 strains was of genotype G3. Genotype G9 was detected in 13% of the Iraqi strains, a similar finding to results in Turkey (*14*). We also detected mixed rotavirus infections in 6% of our patients, again similar to the findings in Turkey (*14*). The presence of mixed rotavirus infections indicates that new rotavirus strains may evolve by reassortment (*1**–**3*).

Finally, among the G9 strains, one P[6]G9 had a long electropherotype, and one P[8]G9 had a short electropherotype. The P[6]G9 and P[8]G9 strains were both cultured and subgrouped by ELISA with monoclonal antibodies and found to be of subgroup II. Partial sequences (831 bp) were obtained for their VP7 genes (AB247941 and AB247943; available from the DNA Data Bank of Japan: http://www.ddbj.nig.ac.jp). They showed 99.4% similarity to each other and >99% similarity to strains from Australia (AY307087), Belgium (AY487858, AY487856), and India (RG9491165). A strain similar to our P[6]G9, called variant 3, was first detected in India, and strains similar to our P[8]G9, called variant 2, have been described in Bangladesh and in the United States (*15*).

Although the major global genotypes (except for G3 strains) were detected, clearly, rotavirus strains are continuing to diversify in Iraq and other parts of the region. This circumstance may pose challenges to the efficacy of rotavirus vaccines.
